# Targeting of phage particles towards endothelial cells by antibodies selected through a multi-parameter selection strategy

**DOI:** 10.1038/srep42230

**Published:** 2017-02-10

**Authors:** Ole A. Mandrup, Simon Lykkemark, Peter Kristensen

**Affiliations:** 1 Department of Engineering, Gustav Wieds Vej 10, Aarhus University, 8000 Aarhus C, Denmark

## Abstract

One of the hallmarks of cancer is sustained angiogenesis. Here, normal endothelial cells are activated, and their formation of new blood vessels leads to continued tumour growth. An improved patient condition is often observed when angiogenesis is prevented or normalized through targeting of these genomically stable endothelial cells. However, intracellular targets constitute a challenge in therapy, as the agents modulating these targets have to be delivered and internalized specifically to the endothelial cells. Selection of antibodies binding specifically to certain cell types is well established. It is nonetheless a challenge to ensure that the binding of antibodies to the target cell will mediate internalization. Previously selection of such antibodies has been performed targeting cancer cell lines; most often using either monovalent display or polyvalent display. In this article, we describe selections that isolate internalizing antibodies by sequential combining monovalent and polyvalent display using two types of helper phages, one which increases display valence and one which reduces background. One of the selected antibodies was found to mediate internalization into human endothelial cells, although our results confirms that the single stranded nature of the DNA packaged into phage particles may limit applications aimed at targeting nucleic acids in mammalian cells.

The vasculature is the main route for transport of molecules in the body. Endothelial cells take part in the formation of new blood vessels through the process of angiogenesis, whose upregulation in tumors is one of the hallmarks of cancer and a major target of cancer therapy. It has been shown that vasculature expresses different antigens depending on the tissue and organ surrounding it, and that distinct antigens are specifically expressed by tumour vasculature^1^,^2^,^3^. Ideally targeted treatment involving the tumor vasculature should target such antigens, however an ideal tumor microenvironment is difficult to mimic *in vitro*, and in this study, the aim is not to target such antigens specifically, but proliferating endothelial cells more broadly.

Many drugs or intervening molecules act on intracellular targets, but active transport is often needed in order for these molecules to be delivered at their site of action. Antibodies with specific targeting and internalizing properties have been explored as a means of specific delivery. Antibody fragments and full size antibodies can be used for targeting drugs by direct coupling or by incorporation into various kinds of nanocarriers, such as lipoplexes and polyplexes^4^. The use of targeting antibodies can improve the specific delivery and reduce the amount of drug needed to obtain the desired effect^5^. Since the pioneering work of Fire and Mello^6^, RNAi has been recognized as a powerful regulator of gene expression, but the obstacle of delivery remains one of the major barriers preventing RNAi from realizing its predicted therapeutic potential^6^,^7^,^8^. Targeted delivery of RNAi by antibodies has been indicated to be a very promising approach that could help to overcome the delivery hurdle while, at the same time, providing cellular specificity^9^,^10^,^11^. In addition to delivery of RNAi, whole genes and other macromolecules can be delivered by targeted immunolipoplexes^12^,^13^.

Within the field of gene therapy, viral delivery is an advantageous therapeutic option, and a variety of eukaryotic viruses have been indicated to have clinical relevance. Of particular interest is the possibility to change the viral tropism using surface-displayed antibodies and other targeting molecules. However, the use of eukaryotic viruses is still controversial and a range of problems needs to be solved before viral therapy can be safely used^14^,^15^.

Prokaryotic viruses, such as the filamentous bacteriophage M13, have been applied in experiments to test phage particles for use as targeted gene delivery vehicles^16^. The phage particle possesses several potential beneficial traits, such as lack of natural tropism for eukaryotic cells, high stability of the phage particles, and convenient production and manipulation^17^,^18^. Targeting of phage particles to relevant cells can be mediated by surface display of antibodies, growth factors, and peptides, and their potential as a drug-delivery platform for cytotoxic small molecule drugs has been examined^19^. Furthermore, phage particles can be used for the delivery of plasmid encoding shRNA against a relevant tumour target^20^. To ensure efficient gene transcription in mammalian cells, adeno-associated virus promoter regions have been introduced to drive the transcription of suicide and reporter genes *in vivo*. This led to reduced tumour size and allowed for tumour imaging when targeted to tumour vasculature^21^.

Several strategies for selecting internalizing antibodies using phage display have been described^22^. When selecting for internalizing antibodies, different temperatures in the incubation and washing steps are used, e.g. a temperature allowing internalization (37 °C) and a non-permissive temperature (4 °C)^23^. Protection of internalized phage particles from lysosomal degradation, e.g. by incubation with chloroquine, is also a commonly employed measure. Internalized phages have traditionally been recovered from the cells by triethylamine (TEA) or detergents, followed by infection into *E. coli* for further propagation (Fig. 1).

In the present study, we aimed to improve selection outcome using a two-step selection strategy with a pre-enrichment for cell surface binding followed by selection for internalization using the pre-enriched library. We further applied different helper phages for the rescue, including the protease sensitive KM13 helper phage, which allows for background reduction in the selection process^24^, and Hyperphage, for increased display level^25^ (Fig. 2). Previously this combination of KM13 and Hyperphage was not used in the same selection strategy to isolated antibodies capable of mediating internalization.

After selection, panels of potentially interesting clones are screened in order to prioritise these clones for further validation. This often entails screening several thousands of clones, and is generally much more time consuming than the selection process itself^26^. When selecting for antibodies mediating a functionality like internalization, this is even more complicated^27^.

The most commonly used screening methods include FACS, immunocytochemistry, and ELISA. Initial screening can be done by detection of the phage particle, as the phage is retained due to its fusion to the displayed antibody. The detection of phage particles can strongly enhance a signal due to their large size and uniformity, which allows the binding of multiple detection antibodies per phage^26^,^28^. When verifying internalization, both co-localization with known internalization markers like transferrin receptors and delivery of GFP reporters to the cytoplasmic space has been described. Additionally, targeting of liposomes has also been applied in order to screen for internalization^18^,^29^.

## Results

### Generation of HMEC-1 cell surface binding sub-libraries

The Tomlinson I, Tomlinson J and Garvan libraries were rescued using either KM13 or Hyperphage, creating 6 initial libraries to be used in selection for enrichment of clones binding HMEC-1 endothelial cells. After selection for binding to HMEC-1 cells, the selection outputs were in the order of 10^4^ to 10^5^ CFU. The selection outputs were again rescued individually using either KM13 or Hyperphage, thus creating 12 sub-libraries enriched for antibody clones binding HMEC-1 cells (Fig. 3A). All the sub-libraries enriched for HMEC-1 binders were evaluated by ELISA against HMEC-1, and CFU output from selection for internalization into HMEC-1 was determined (Fig. 3B & C).

For the internalization selection, a procedure for removal of extracellular phages was established. First, phage incubations were made at 4 °C to prevent internalization. After 7 washes with PBS, the number of phage particles present in the wash solution was at a steady low level, according to CFU. By performing an additional acidic wash (pH 2.2), followed by trypsin treatment, more phage particles could be removed from the cells, ultimately resulting in a remaining output of background phage particles that was roughly 10^10^ times lower than the input (data not shown).

For selections for internalization, the temperature was increased to 37 °C, with an incubation time of 1½ hours, to improve the chances of selecting antibodies binding rapidly internalizing antigens.

Different methods for retrieval of phage particles from HMEC-1 cells after selection for internalization were tested. The results showed that freeze/thaw cycles of cells in pure deionized water gave slightly more infective phage particles after selection for internalization than did cell lysing with either 0.25% CHAPS, 0.25% TritonX100, 0.1 M TEA or 1% TritonX100 (data not shown).

### Selection for internalization using enriched libraries

To further test the effect of combined rescue with helper phages, six separate selections for internalization were made using the six sub-libraries from Tomlison I, Tomlinson J and Garvan generated by combined rescue with Hyperphage and KM13 helper phages. From each of the six sub-libraries, 2 × 10^8^ phage particles were used, in order to fully cover the possible diversity in the sub-libraries while reducing biased selection for highly abundant cell surface antigens. The selections resulted in an output of around 10^4^ CFU. From each selection, 96 clones were picked and grown in 96 well-plates.

Phage particles were produced from six 96-well plates and tested in ELISA. The clones were screened in duplicate for binding to growth medium and HMEC-1 cells. Several phage antibodies with affinity for media were observed; in particular, the Tomlinson sub-libraries contained an especially large number of growth media binders (Table 1).

Clones binding HMEC-1 cells were further tested in ELISA against other cell lines to determine their specificity for endothelial cells. ASF-2 human fibroblasts and hMSC human bone marrow stromal cells were used for the screening. The ELISAs were made with phage particles produced in 96-well plates rescued using Hyperphage (Fig. 4). To validate the whole cell ELISA procedure with phage particles, fourteen clones were selected and a whole cell ELISA was made using equal amounts of phage particles rescued using either KM13 or Hyperphage. All clones were tested in duplicate with each helper phage to test the effect of display level on ELISA signal, and the signal was normalised to the cell number based on Janus green staining (Fig. 5).

Eight of the fifteen clones from the Garvan library verified to bind HMEC-1 cells and showing a high degree of specificity were further shown to bind HUVEC endothelial cells, indicating a preference for endothelial cells in general (data not shown). Sequencing of the eight clones revealed that three clones had amber stop codons and two contained paired cysteine residues in the CDR3 region. Soluble antibody fragments without fusion to the phage protein III were expressed and purified from the 5 clones without stop codons.

Soluble antibody was incubated with live HMEC-1 cells to further validate the ability to mediate internalization (Fig. 6). A clearly punctate staining pattern was observed, further supporting internalization into endosomes (only data for H8 shown). The clones containing cysteines tended to be more prone to forming aggregates and were not taken further. Of the remaining 3 clones, H8 gave the best yield of soluble antibody and was therefore chosen for further analysis.

To confirm the location of the antibody within the cells, Alexa-568 conjugated transferrin was incubated along with the antibody H8 (Fig. 6). The staining indicates a co-localization between H8 and transferrin in the cells.

### Gene delivery

Live HMEC-1 cells were incubated with the H8 phage clone in order to allow for internalization at different concentrations (Fig. 6). Only very weak staining was observed when 10^9^ phage particles was added, while addition of 10^11^ phage gave a clearly visible staining. The KM13 helper phage was used as negative control and did not give any visible staining, even at the highest phage concentration. The altered staining pattern obtained when cells are fixed in PFA before incubation with phage further indicates that internalization mediated by the antibody is occurring (Fig. 6).

In order to examine the ability of the phage to deliver a gene into eukaryotic cells when displaying the H8 antibody on the surface of the phage particle, the gene encoding the H8 antibody was sub-cloned into the pFROG plasmid containing a GFP reporter. The pFROG-H8 phagemid was rescued using either KM13 or Hyperphage. The phage particles were incubated with live HMEC-1 cells for 24 or 48 hours before cells were screened for GFP expression. There was, however, no detectable expression of GFP in any of the cells (data not shown). To detect for the actual presence of phage particles, the cells were fixed, permeabilised and stained for phage particles (Fig. 7). The staining clearly indicates that phage particles were present in the cells. To enhance the efficiency of phage gene delivery, camptothecin (CPT) was added, as it has previously been reported to enhance expression of GFP from single stranded DNA^30^. There was, however, no visible effect of CPT on gene expression efficiency (data not shown).

Transfections with single stranded pFROG-H8 DNA isolated from phage particles and the corresponding double stranded pFROG-H8 plasmid DNA isolated from bacteria were performed to evaluate the actual efficiency of expression of proteins in the cells. The DNA was transfected into various cell types and the percentage of GFP expressing cells were estimated. When transfecting with ss-DNA, less than one in a thousand cells showed GFP expression. It was estimated that 15–20% of the total HMEC-1 cells were expressing GFP after transfection with ds-DNA (Fig. 8). MCF-7 cells have previously been reported to sustain expression when infected by phage particles, so transfections with ss-DNA and ds-DNA were performed using this cell line^18^. It was estimated that 15–20% of the MCF-7 cells were expressing GFP after transfection with ds-DNA and 3–5% after transfection with ss-DNA (Fig. 8C,D). It was noteworthy that the MCF-7 cancer cell line had much smaller differences in expression of GFP after transfection with ss-DNA and ds-DNA when compared to the HMEC-1 cells. HUVEC, ASF-2 and HEK293 cells were likewise transfected with ss-DNA and ds-DNA (Fig. 8). Transfection results using these cell lines were those obtained for HMEC-1 cells with regards to the difference between GFP expression levels from ss-DNA and ds-DNA. Furthermore, for the non-cancer cell lines, there was a general tendency towards more dead cells in the wells transfected with ss-DNA, than was the case in the wells transfected with ds-DNA. For the MCF-7 and HMEC-1 cell lines these results were reproduced using a plasmid containing a humanized GFP gene from R. *reniformis* (Supplementary Figure 1).

## Discussion

Receptor dimerization is often an essential first step for receptor internalization. For this reason, polyvalent display systems could be envisioned to be superior to monovalent display formats for selecting antibodies which bind receptors and become internalized^31^. Even so, several groups have reported successful selections for internalizing antibodies using the monovalent phagemid system^32^,^33^. When a phagemid is rescued using a helper phage where gIII has been deleted, it is possible to force polyvalent display into the phagemid format. Different gIII-negative helper phages exist, although comparisons indicate that Hyperphage might be the most effective in this regard^34^.

The frequency of antibody clones in a library that will be able to bind a surface antigen and then trigger internalization is going to be very low. It could therefore be an advantage to use a two-step procedure where an enrichment strategy initially selects for antibodies binding cellular surface antigens, followed by selecting the resulting panel of cell binding antibodies for the ability to mediate internalization.

Most selections for internalizing antibodies targeting specific cell types have made use of various depletion strategies to ensure that the selected antibodies binds antigens specifically expressed by certain cell types. However, depletion can be problematic. For example, if the aim of a selection is to isolate antibodies capable of delivering drugs to tumor cells *in vivo*. In this regard Gillet *et al*. have previously shown that cultured cancer cell lines resemble each other more than the clinical sample regardless of tissue of origin^35^. Furthermore, depletion will only remove the phage particles which have bound to the target cell through an antigen-antibody interaction, and not the bulk of phage particles, which bind to the target cells through non-specific interactions. Finally, as depletion relies on shifting the equilibrium from the target cell to the non-target cells, this will depend on affinity and concentration of target antigen on the two cell populations, and it is therefore difficult to ensure that depletion is 100% effective^36^.

In this study we use “pre-selection” for enrichment of surface binders combined with packaging of phage particles using the trypsin-cleavable KM13 helper phage, which will help to reduce the background while still allowing selection for higher affinity and specificity^37^; we have not included a depletion step.

In many studies, multiple rounds of selection are used. Unfortunately, the use of multiple rounds of selection reduces diversity not only due to selection for binding, but also due to different growth advantages between clones, causing an undesired loss of diversity^38^,^39^. Clones which are highly abundant in the library from the start and clones binding a highly abundant or highly accessible antigen will also result in an undesired bias^36^. Finally, multiple selection rounds favor high affinity clones; however this may not always be the most desirable clones for targeting and delivery^40^.

**Figure 2:**
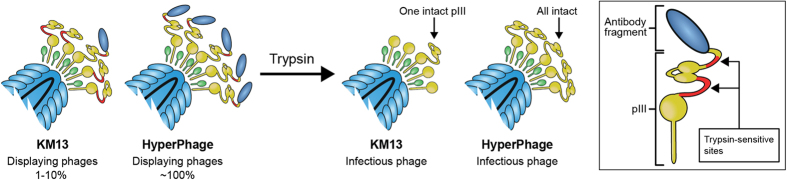
Comparing helperphages with different properties. Functionalized helper phages like the KM13 and the Hyperphage have been developed for rescuing phagemids into phage particles. Normal phagemid rescue results in only 1–10% of phage particles displaying a single antibody fragment. When rescuing phagmids with KM13 helperphage the trypsin cleavage site between domain 2 and 3 of pIII results in the non-displaying pIII from the helper phage being rendered non-infective. PIII fused with antibody encoded by the phagemid retains infectivity. Hyperphage is deleted in the gene encoding pIII so that no pIII can be derived from the helper phage, which in theory leads to 100% antibody display. The background can, however, not be removed when using Hyperphage.

Based on these considerations, only two rounds of selection were made, with an initial selection for enrichment of binders to surface molecules of HMEC-1 cells, followed by a selection for internalization. We used a combinatorial packaging strategy to see how the benefits of KM13 (lower background) or the benefits from Hyperphage (polyvalency) would influence the selections (Fig. 2).

**Figure 3:**
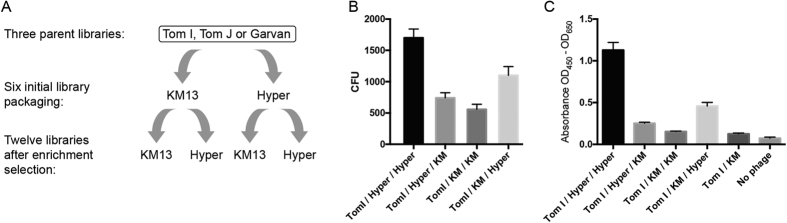
Selection scheme and initial effect on selection output. (**A**) Three different phagemid based parent libraries i.e. Tomlinson I, Tomlinson J and Garvan were each packaged into phage particles using either the gIII deficient Hyperphage or the protease sensitive KM13. The resulting six libraries were used in selections on HMEC-1 cells for surface binders and the resulting phage output from the selections were again packaged using either KM13 or Hyperphage as helper phage resulting in a total of twelve different sub-libraries. Selections using phage packaged with different combinations of helper phage were used for evaluating the effect of the helper phage derived characteristics on internalization. (**B**) The effect of using either Hyperphage or KM13 for rescue of phage particles was evaluated. The results showed a clear effect on the output of phage particles after selection for internalization, with Hyperphage packaging generally resulting in more CFU than KM13. (**C**) Selections for internalization were made on HMEC-1 cells using the Tom I parent library or one of four Tom I sub-libraries enriched for antibodies binding HMEC-1 surface antigens. HMEC-1 cells were fixed and permeabilized for ELISA. All enriched sub-libraries give a signal above the unselected parent library. Again a clear difference in signal between the sub-libraries with higher signal from the sub-libraries packaged with Hyperphage after the selection for enrichment is evident. Only results for the Tomlinson I libraries are shown.

After the initial pre-enrichment of the libraries for antibodies binding to surface antigens on HMEC-1 cells, the different libraries were used in selections for internalization. Here, the number of ampicillin resistant bacterial colonies obtained after infection with phages from cell lysate showed a distinct trend towards a higher number of colonies from the sub-libraries rescued with Hyperphage compared to KM13 (Fig. 3B). Using the sub-libraries in a whole cell ELISA, a distribution in signal similar to that observed in CFU output is apparent (Fig. 3C). This may indicate that the effect of Hyperphage packaging is caused by less efficient removal of extracellular phage from the cell surface, rather than from internalization, since the cells have not been permeabilized for the ELISA. It is difficult to determine what the exact contribution to the results is from the reduction of extracellular phage due to packaging with KM13 versus the increase of internalized phage due to packaging with Hyperphage. The matching results between CFU count and ELISA, however, indicates that Hyperphage packaging primarily increases output from both assays by increasing bound phages to the extracellular antigens.

**Figure 4:**
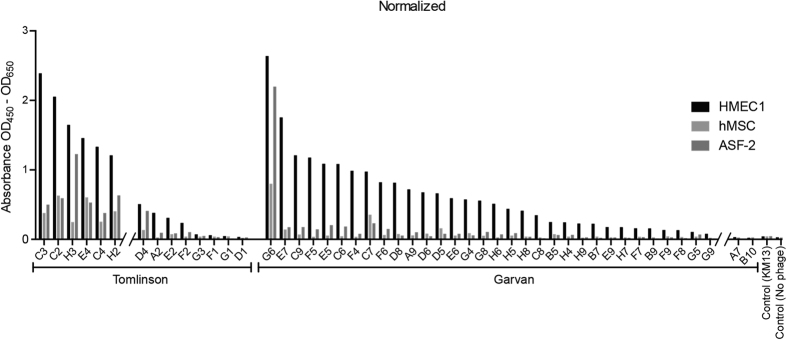
ELISA validation of cellular specificity. ELISAs were made to test the cell type specificity of the cherry picked clones. Each clone was tested for binding to HMEC-1 cells, ASF-2 fibroblasts and hMSC bone marrow stromal cells in parallel. The results have been split into clones originated from the Tomlinson I & J libraries (left) and clones from the Garvan library (right). The ELISAs were made with phage particles rescued with Hyperphage.

One 96-well plate from each selection for internalization with the different sub-libraries was screened for binding to HMEC-1 cells in phage ELISA (Table 1). As no depletion step was applied, there were a high percentage of antibodies binding components of the cell media coated onto the plastic surfaces of the culture plates. All antibodies were tested in parallel on EGM and HMEC-1 cells, and with phage-antibody from same batch to improve the congruency between EGM positive and HMEC-1 positive clones (Table 1). The number of cell binding antibodies was notably higher from the Garvan library than from the Tomlinson libraries, which could either be due to the higher diversity of the Garvan library or the smaller size of the dAbs, improving their access to cryptic antigens. Screening the HMEC-1 positive antibodies for binding to other cell lines further indicated that the dAb Garvan clones seem to be more cell-type specific than the scFv Tomlinson (Fig. 4).

**Figure 5:**
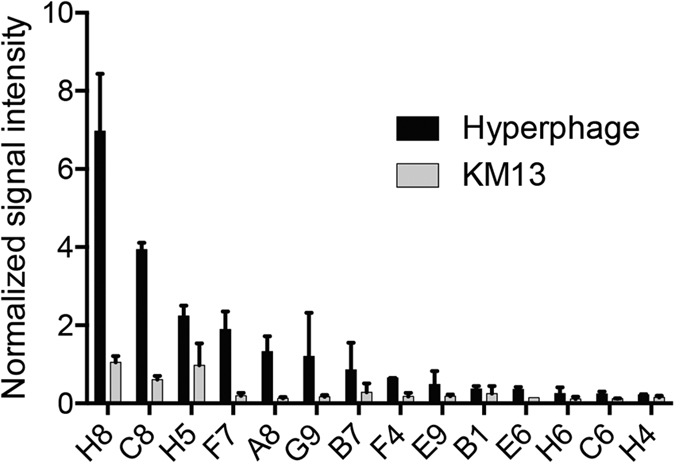
Comparing helperphage effects on ELISA signals. ELISA was made on HMEC-1 cells using 15 cherry picked clones. The clones were rescued with KM13 and Hyperphage to compare the effect of the helperphage on the ELISA signal. The results were normalized based on cell number measured by Janus green staining.

To further validate the phage ELISA on whole cells, fourteen phage-antibodies were tested in an ELISA where both the number of cells per well and the number of phage particles from the individual rescues were taken into account (Fig. 5). These results further indicate that there is an avidity effect from Hyperphage and that this effect could be advantageous in phage ELISA with regard to detection of low affinity binders and clones binding rare antigens. Comparing signals in whole cell ELISA after normalizing signal to cell numbers by Janus green staining, we can conclude that the number of cells left in the wells is high enough to ensure trustworthy ELISA results (data not shown). The valency of the antibody displayed on the phage seems to affect the results more, as can be seen by the higher signal from clones rescued with Hyperphage as compared to KM13.

**Figure 6:**
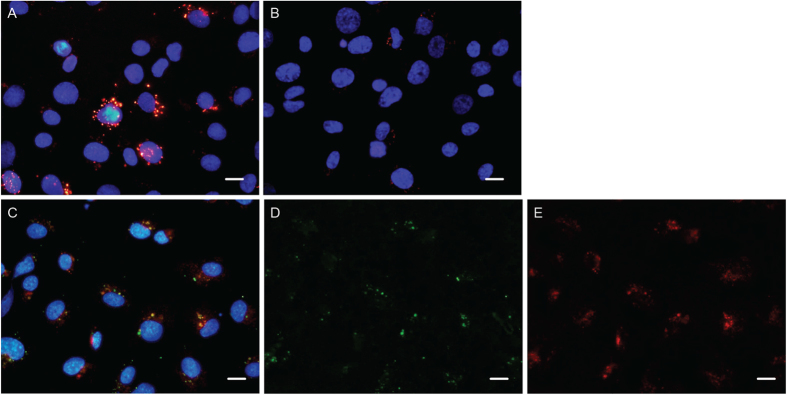
Staining HMEC-1 cells to detect internalization. (**A**,**B**) HMEC-1 cells incubated with either soluble H8 antibody or secondary antibody only. Incubations were made under internalizing conditions and followed by fixation, permeabilization and staining of internalized antibody (red). Cell nuclei are stained by dapi (blue). **(C**–**E**) HMEC-1 cells incubated with transferrin (red) and H8 antibody (green). Incubations were made under internalizing conditions and followed by fixation and permeabilization to stain H8. Scale bars = 10 μm.

Based on the ELISA and sequencing results, eight clones were expressed as soluble antibodies. Of these eight clones, H8 was chosen for more in depth analysis. Fluorescent staining with phage-antibody H8 showed that the clone binds endothelial cells and seems able to internalize (Fig. 6).

The punctate spots observed in the staining indicate that the H8 antibody was internalized into endosomes. The localization of internalized antibody was also to a large extent seen to overlap with transferrin in the cells, further substantiating H8’s entry into the endosomes (Fig. 6).

It has been suggested that the phage particle itself could be used as a vehicle for drug delivery. The biggest hurdle for the use of phage particles for delivery of nucleic acid drugs is probably the intracellular destiny of the phage and the low efficiency of transcription from single stranded DNA (ssDNA) in mammalian cells. Previously, it was shown that even though almost all cells had internalized targeted phage particles, expression from a GFP reporter in the phage genome was quite low^17^,^18^.

**Figure 7:**
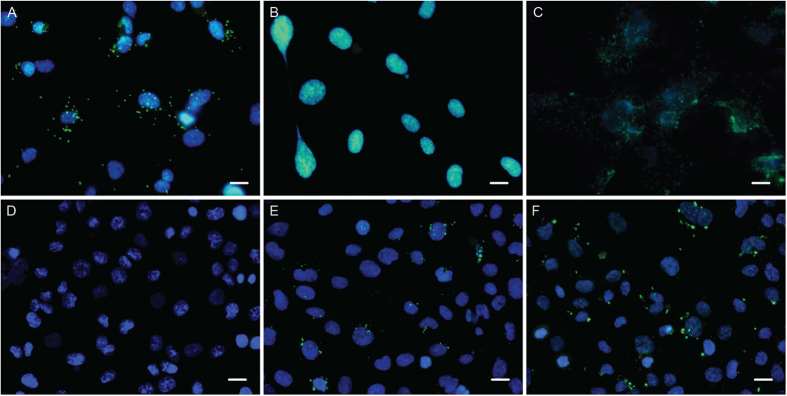
Tracking antibody-phage internalization by ICC. HMEC-1 cells incubated with either non-displaying KM13 helper phage (green) or phage displaying the H8 rescued with KM13 (green). Incubations were made under internalizing conditions and followed by permeabilization and staining of phage particles. (**A**) Cells incubated with 10^11^ H8 displaying phages pr. well. (**B**) Cells incubated with 10^11^ non-displaying KM13 helper phage. (**C**) Cells fixed in PFA and incubated with 10^11^ H8 displaying phages pr. well. (**D**) HMEC-1cells incubated 48 hours without phage or with 5 × 10^11^ H8-pFROG rescued with Hyperphage (**E**) or KM13 (**F**). The cells were washed, fixed and permeabilized before staining of phage particles (green). Cell nuclei are stained in blue. Scale bars = 10 μm.

We tried to use expression of GFP, delivered to HMEC-1 cells by phage particles packaging the pFROG GFP reporter and targeted by display of the selected H8 antibody, as an indicator of internalization. Internalization of H8 targeted phage particles was visible in HMEC-1 cells incubated at 37 °C and showed a markedly different staining pattern compared to cells incubated at 4 °C (Fig. 7A–C). The effect of the valence was also tested by comparing phage particles displaying H8 rescued with either Hyperphage or KM13. There was no clear difference, which may indicate that receptor dimerization is not necessary for internalization (Fig. 7D–F).

**Figure 8:**
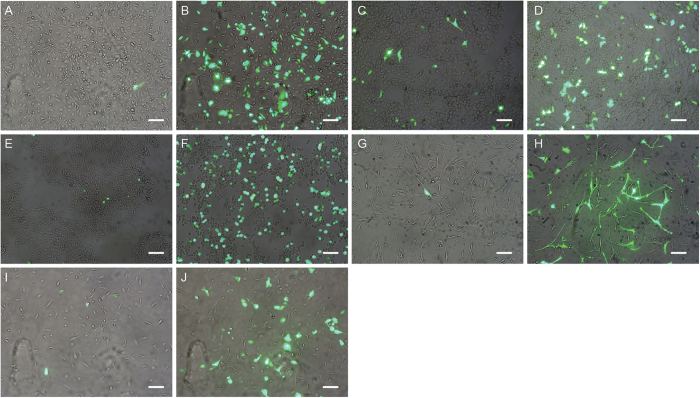
Expression efficiency from ss-DNA versus ds-DNA in various cell lines. Cell lines were transfected using either single or double stranded form of the GFP encoding 8H-pFROG plasmid (green) and imaged with phase contrast to visualize non-fluorescent cells. HMEC-1 cells transfected with ssDNA (**A**) and ds-DNA (**B**). MCF-7 breast cancer cells transfected with ssDNA **(C)** and with ds-DNA **(D)**. HEK293 cells transfected with ssDNA (**E**) and with ds-DNA (**F**). ASF-2 cells transfected with ss-DNA **(G)** and dsDNA **(H)**. HUVEC cells transfected with ss-DNA **(I**) and ds-DNA **(J)**. Scale bars = 1 μm.

There was no indication of GFP expression after 48 hours, even though staining for the phage particles showed that the phage was present within the cells. Previously, great differences in transduction efficiencies between different cell types by targeted phage particles has been reported, possibly due to the single stranded nature of the phage DNA^15^,^29^. We therefore tested the expression efficiency after transfection with ss-DNA and double stranded DNA (ds-DNA) of pFROG into endothelial cells. The results did indeed indicate that expression of GFP after transfection with ss-DNA was significantly lower than after transfection with ds-DNA. The same was observed for a number of non-cancerous cell lines, and significant expression from ss-DNA could only be observed for the MCF-7 breast cancer cell line (Fig. 8). The incubation of HMEC-1 cells with CPT after transfection did not improve expression, although this had been reported to increase expression from ss-DNA^30^. It could be speculated that certain cancer cell lines might harbor mutations that will allow them to use ss-DNA more effectively. Whether this would show to have relevance *in vivo,* however, needs to be investigated. An approach to improve transduction efficiency of mammalian cells with ss-DNA could be to construct complementary sequences making the ss-DNA capable of forming double stranded stretches. This has previously been reported to increase expression from ss-DNA^41^.

Other factors are also important for transduction efficiencies of mammalian cells using phage particles. For instance, internalized phage particles have to exit the endosomes and break apart to release their DNA. It has been suggested that endosomal escape could be mediated by incorporating histidine residues in the phage coat proteins, acting as a proton sponge and causing osmotic rupture of the late endosomes^18^.

In this report, we investigate the use of novel combinations of selection strategies for selecting internalizing antibody clones by phage display, and we validate the internalization of a selected clone into endothelial cells. Our data further indicates that measures can be taken to reduce the variability seen with phage ELISA on whole cells by using Hyperphage for phage rescue, thereby improving the chance of detecting weak binders and clones binding rare antigens after selections. We also confirm previous observations that ss-DNA is very inefficiently processed in mammalian cell lines, however, whether cancer cell lines might be more promiscuous in the use of ss-DNA needs to be further investigated.

## Methods

### Biopanning of HMEC-1 cells for surface binding sub-libraries

TG1 and HB2151 (Source Bioscience) *E. coli* strains were used for phage production and XL-1 blue were used for cloning (Agilent Technologies). Tomlinson I and J scFv libraries (Source Bioscience) contain approximately 1.47 × 10^8^ and 1.37 × 10^8^ clones respectively^42^. The Garvan (dAb) library contains approximately 1.6 × 10^10^ clones and was kindly supplied by Dr. Daniel Christ, Garvan Institute, Sydney, Australia.

Selections for libraries enriched for surface binders to HMEC-1 cells were made on sub-confluent HMEC-1 cells grown in T_75_ bottles. Cells were moved to 4 °C, 20 minutes prior to selection to prevent internalization of phage particles. The phage libraries used for selections were incubated in media coated bottles to subtract media binders from before selection on cells. Approximately 1 × 10^11^ infective phage particles were added to media coated bottles and incubated half an hour in 5 mL EGM. The subtracted phage library was then added to the HMEC-1 cells and incubated for 1 hour at 4 °C. The cells were washed 3 times in cold EGM and 4 times in cold PBS, using 10 mL per wash. The phage particles were eluted with 2 mL freshly prepared trypsin 1 mg/mL at 37 °C. The trypsin treated cells were spun down and cell pellets were lysed by osmotic swelling and freeze/thaw cycling. The supernatant and the cell lysates were mixed with TG1 bacteria at an OD_600_ of 0.4. The mixtures were incubated 45 min. at 37 °C. The bacteria were plated on agar-plates containing ampicillin and 1% glucose and incubated at 30 °C overnight. Colonies were scraped off and used for production of new sub-libraries, rescuing the phagemid using either Hyperphage or KM13 helper phage.

### Selection for internalization

Prior to the incubation with phage, 100 μM chloroquine (Sigma-Aldrich) was added to the media of live HMEC-1 cells and incubated for 45 minutes to protect internalized phages from entering the lysosomes. Phages were added to the cells and incubated 1½ h. at 37 °C, followed by 2 washes in cold EGM and 2 washes in cold PBS. The cells were then incubated in cold 0.2 M glycine pH 2 for 10 minutes followed by a wash in cold PBS with 0.05% Tween20 and 2 washes in cold PBS. Finally, the cells were trypsinized for 20 minutes at 37 °C and spun down for 25 minutes at 400 g. The cell pellets were resuspended in 0.5 mL deionized H_2_O with 5 mM EDTA and 3 freeze/thaw cycles were made. The cell lysate were mixed with 5 mL TG1 of OD_600_ = 0.4 for 45 minutes at 37 °C. The bacteria were then plated on agar plates containing ampicillin and 1% glucose and incubated overnight at 30 °C.

TritonX100 (Sigma-Aldrich), triethylamine (TEA) (Sigma-Aldrich), CHAPS (Sigma-Aldrich) and osmotic swelling followed by freeze/thaw cycles were tested for optimal cell lysis. Selections on HMEC-1 under internalizing conditions were made, the cells were trypsinized and lysed using either 0, 25%, 1% TritonX100, 0, 1 M TEA, 0, 25% CHAPS or osmotic swelling followed by freeze/thaw cycles. When TEA treatment was used, the mixture was neutralized with 1 M Tris-HCl pH 7.5 before infection into TG1.

Phage particles were produced from *E. coli* bacteria grown in appropriate media as described in the Tomlinson library protocol (Source Bioscience). Phage particles were produced using either the protease sensitive helper phage KM13^24^ or the gene III deficient helper phage Hyperphage^25^ (Progen Biotechnik).

Phages were either titrated by counting colony forming units (CFU) or from plates estimated from absorbance at 269 nm and 320 nm as described in Barbas *et al*.^43^.

### Expression and purification of soluble antibody

Expression of soluble antibody were made from bacterial cultures by induction with IPTG at OD_600_ = 0.7–0.9. Cultures were incubated overnight at 37 °C after induction.

Antibodies were purified from cleared supernatants by either Protein A HP spin traps (GE Healthcare) or immobilized metal ion affinity chromatography (IMAC) His spin traps (GE Healthcare).

### Mammalian cell culturing

HMEC-1: Immortalized human micro vascular endothelial cells (ATCC). HUVEC: Human umbilical vein endothelial cells (Lonza). HMEC-1 and Huvec cells were cultured in Endothelial Growth Medium (Lonza). hMSC: Immortalized human bone marrow stromal cells^44^, kindly supplied by Professor Suresh Rattan, Department of Molecular biology and Genetics at Aarhus University. hMSC cells were cultured in DMEM with 10% FCS. HEK 293: Human embryonic kidney cells (ATCC, USA). HEK293 cells were cultured in DMEM with 10% FCS.

ASF-2: Primary human skin fibroblast cells. ASF-2 cells were cultured in DMEM with 10% FCS. ASF-2 cells were kindly supplied by Professor Suresh Rattan Institute of Molecular biology and Genetics at Aarhus University. MCF-7: Human luminal epithelial breast cancer cell line (ATCC). MCF-7 cells were cultured in DMEM with 10% FCS and 0.01 mg/mL insulin.

All plasticware used for cell culturing were from Corning. All cells were grown at 37 °C with 5% CO_2_ and high humidity and passaged when 80% confluent.

### ELISA screening

ELISA wells were coated for a minimum of 2 hours with antigen and blocked using 2–4% mPBS for a minimum of 1 hour before incubation with phage antibody clones. Phage clones were incubated minimum 2 hours with antigen before washing 6 times with 200 μl PBS per well. Mouse anti-M13 HRP conjugated antibody (GE Healthcare) diluted 1:4000 in 2% mPBS were used for detection. ELISA wells were washed 3 times in 200 μl PBS, and incubated with 100 μl TMB readymade solution (Kem-En-Tec Diagnostics). The reaction was terminated by adding 50 μl 1 M H_2_SO_4_ and the result was read in an ELISA plate reader at 450 nm with subtraction of 650 nm background reading.

When ELISA was made using free soluble antibodies, the washing procedure was reduced to two washes before incubation with detection antibody.

### Whole cell ELISA screening

For ELISA screening on whole cells, the cells were seeded a day before the ELISA in sterile flat bottomed 96-well culture plates. The number of cells seeded per well were between 15,000–25,000. Cells were washed one time in sterile DPBS before the ELISA procedure (as described above).

In some experiments Janus green (Thermo scientific) was used for quantification of cells after ELISA development^45^. Here, the cells were rinsed 3 times in H_2_O and incubated with 40 μl 0.1% Janus Green per well for 10 minutes. The cells were then rinsed 3 times in H_2_O (100 μl per well) and 100 μl 0.5 M HCl were added and incubated for 10 minutes before the plate was read in an ELISA plate reader at 595 nm.

### Fluorescence microscopy

For staining of internalized phage particles on live cells, approximately 70,000 HMEC-1 cells were seeded per well in a 4-well permanox chamber slide a day in advance. One hour prior to addition of phage 100 μM chloroquine (Sigma-Aldrich) was added to the media. The cells were moved to room temperature and phage or soluble antibody was added directly to the media and incubated for 30 minutes. The cells were washed one time in fresh medium and moved to 37 °C for 2 hours. After incubation the cells were washed 3 times with EGM and 3 times with PBS and fixed in 2% PFA for 10 minutes at room temperature, residual PFA was removed by PBS. The cells were permeabilized using 0.1% TritonX100 for 15 minutes at RT. The permeabilized cells were blocked 1 hour in 2% mPBS. The cells were incubated 2 hours with mouse anti-M13 antibody (GE Healthcare) (1:4000) in 2% mPBS or anti-Myc-Cy3 (Sigma-Aldrich) (1:1000) For phage staining anti-M13, this was followed by 2 times wash in PBS and incubation with alexa-488 conjugated goat anti-mouse antibody (GE Healthcare) (1:500) in 2% mPBS for minimum 1 hour. The cells were washed 2 times in PBS and mounted using Vectashield mounting media (Vectorlabs).

### Phage particle gene delivery

The pFROG plasmid was kindly supplied by Professor Jim Marks (Departments of Anesthesia and Pharmaceutical Chemistry University of California, San Francisco, USA). H8 antibody clone was sub-cloned into pFROGby Sfi I and Not I. The resulting H8-pFROG was packaged into phage particles displaying H8 from pIII using either KM13 or Hyperphage. Approximately 30.000 HMEC-1 cells were seeded in each well and grown overnight and 5 × 10^11^ H8- pFROG phage were added to each well in fresh EGM media. Cells were surveyed for GFP expression after 24 and 48 hours. For detection of internalized phages, the cells were washed 2 times in PBS, fixed in 10 minutes in 2% PFA and permeabilized in ice cold 99% methanol. Cells were blocked 1 hour in 2% mPBS and incubated overnight with mouse anti-M13 antibody (1:1000) in 2% mPBS. The cells were washed 2 times in PBS and incubated 2 hours with alexa-488 conjugated anti-mouse antibody (1:500). The slide were washed 3 times in PBS and mounted with Vectashield.

To test the effect of camptothecin (CPT) (Sigma-Aldrich) CPT was added to a final concentration of 400 nM, 1 μM or 10 μM to the cells and incubated for 7 hours before the media was exchanged for fresh EGM. The cells were then incubated and surveyed for GFP expression after 48 hours.

### Transfections

Pfrog-H8 and vitality hrGFPII-C (Agilent) DNA was used for transfections. Single stranded DNA was extracted from PEG6000 precipitated phage particles by phenol extraction using phase lock gel tubes (5Prime). Double stranded DNA was purified from *E. coli* cultures using plasmid DNA purification kits (Thermo scientific).

Approximately 80,000 cells were seeded per well in 12-well plate in complete media except for antibiotics and grown overnight. The cells were approximately 80% confluent the following day. For each well 3 μl of lipofectamin 2000 (Thermo scientific) were mixed with 100 μl media and 1 μg of DNA were mixed into 100 μl media. The Cells were given 1 mL fresh basal media. After 5 minutes the lipofectamin 2000 and DNA were mixed together and incubated 30 minutes at RT. The lipofectamin/DNA mixtures were added to the cells and incubated 5 hours in 37 °C. Fresh media were added to the cells and they were incubated overnight at 37 °C. To test the effect of CPT the same procedure was used, except that CPT were added after ca. 20 hours to a final concentration of either 50 nM, 500 nM, 2 μM or 10 μM, the cells were then incubated for 7 hours before the media was changed. The cells were then incubated overnight before surveyed for GFP expression.

## Additional Information

**How to cite this article**: Mandrup, O. A. *et al*. Targeting of phage particles towards endothelial cells by antibodies selected through a multi-parameter selection strategy. *Sci. Rep.* **7**, 42230; doi: 10.1038/srep42230 (2017).

**Publisher's note:** Springer Nature remains neutral with regard to jurisdictional claims in published maps and institutional affiliations.

## Supplementary Material

Supplementary Figure 1

## Figures and Tables

**Figure 1:**
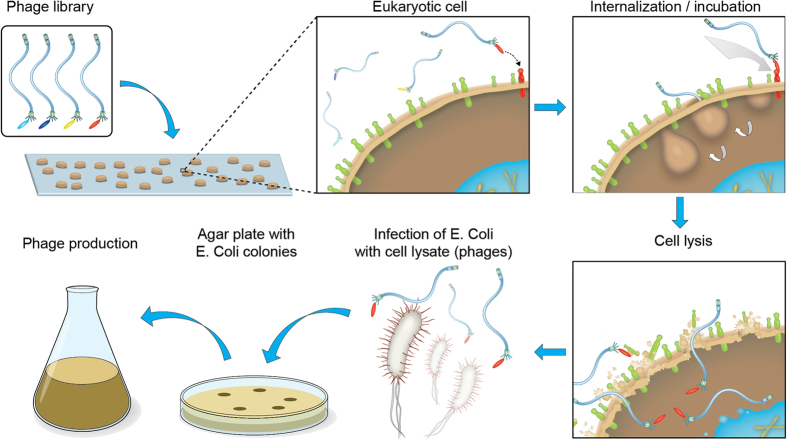
Schematic selection for internalization. In a basic selection for internalization the phage library is incubated with the live cells at 37 °C in order to allow internalization to happen. Washing steps are performed to remove the library clones not internalized. The cells are then lysed to release the internalized phage and the lysate is mixed with *E. coli* for infection. The bacteria surviving (due to phage encoded antibiotic resistance) on selective agar plates containing antibiotics can be used for production of new phage particles for additional rounds of selection or for screening.

**Table 1:** Selection outputs.

Sub-libary	Number of clones giving signal on HMEC-1 cells in ELISA
Tom I/KM/Hyper	2
Tom I/Hyper/KM	9
Tom J/KM/Hyper	11
Tom J/Hyper/KM	7
Gar/KM/Hyper	24
Gar/Hyper/KM	22

From each of six different selections for internalization into HMEC-1 cells 96 clones were picked and screened in ELISA. The clones were screened for signal against HMEC-1cells and the EGM medium used for cultivating the HMEC-1 cells. The EGM positive clones were subtracted from the HMEC-1 positive clones to give the number of clones only specifically binding HMEC-1 cells. Only clones shown to be specific for HMEC-1 in duplicate ELISAs were counted as positives.
