# Supplementary material for: Targeting of phage particles towards endothelial cells by antibodies selected through a multi-parameter selection strategy
Source: Sci Rep. 2017 Feb 10;7:42230. doi: 10.1038/srep42230 (PMC5301479; doi:10.1038/srep42230)
Supplement: Supplementary Figure 1 [file srep42230-s1.pdf]

Supplementary material:

Targeting of phage particles towards endothelial cells by antibodies selected through a multi-parameter selection strategy

Ole A. Mandrup[1], Simon Lykkemark[1] and Peter Kristensen[1*]

[1] Department of Engineering, Gustav Wieds Vej 10, Aarhus University, 8000 Aarhus C, Denmark

*Corresponding author: Peter Kristensen, Department of Engineering, Aarhus University, Denmark, pk@eng.au.dk

Supplementary figure 1

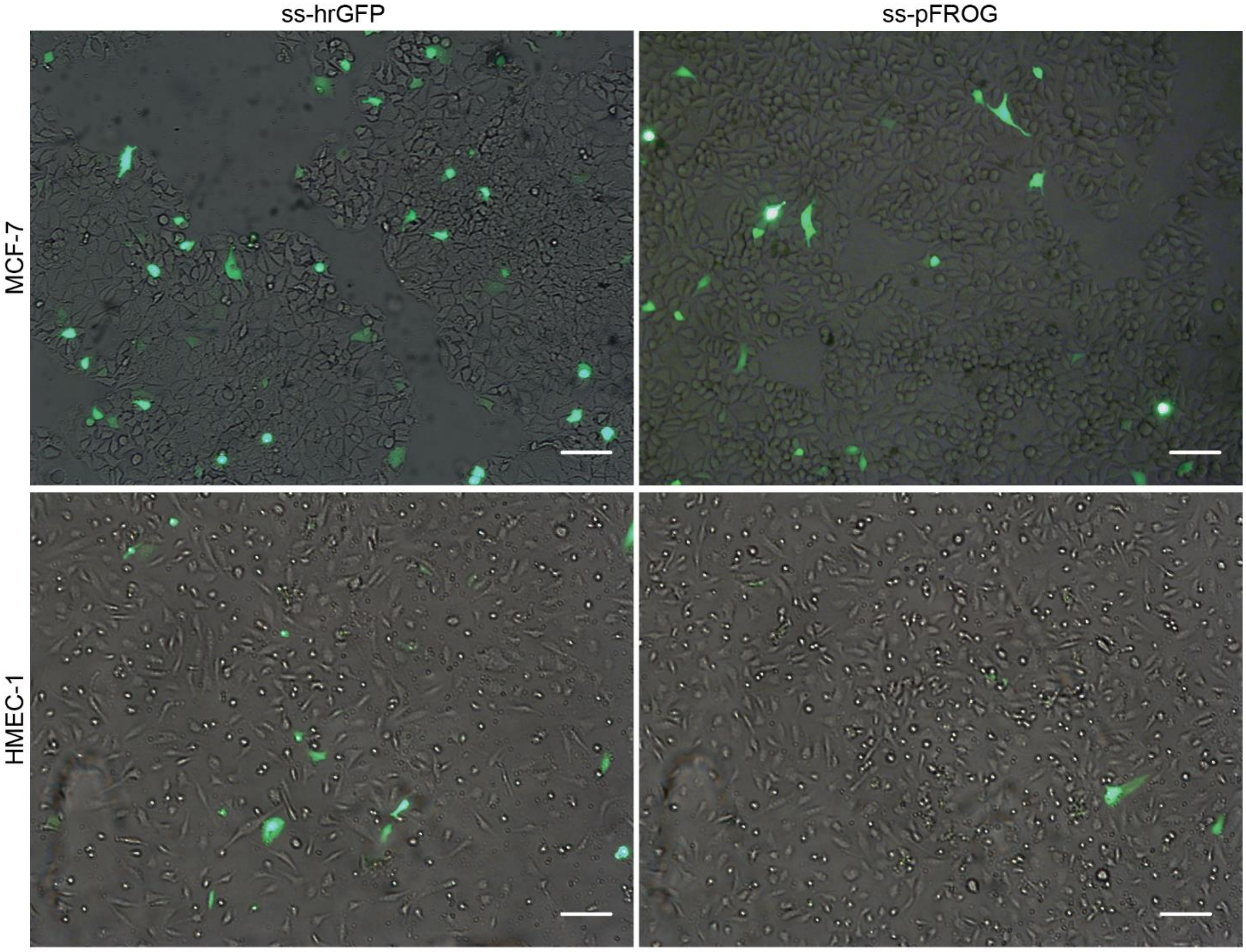

Supplementary figure 1: MCF-7 and HMEC-1 cell lines were transfected using single stranded forms of GFP encoding plasmids H8-pFROG and vitality hrGFP-II. The transfection results for ss-pFROG and ss-hrGFP DNA are shown in green with the total number of cells imaged with white light in phase contrast. Scale bars = 1 μm.
